# Viewing Pictures of a Romantic Partner Reduces Experimental Pain: Involvement of Neural Reward Systems

**DOI:** 10.1371/journal.pone.0013309

**Published:** 2010-10-13

**Authors:** Jarred Younger, Arthur Aron, Sara Parke, Neil Chatterjee, Sean Mackey

**Affiliations:** 1 Department of Anesthesia, Stanford University School of Medicine, Stanford, California, United States of America; 2 Department of Psychology, State University of New York at Stony Brook, Stony Brook, New York, United States of America; 3 Department of Human Biology, Stanford University, Stanford, California, United States of America; Mount Sinai School of Medicine, United States of America

## Abstract

The early stages of a new romantic relationship are characterized by intense feelings of euphoria, well-being, and preoccupation with the romantic partner. Neuroimaging research has linked those feelings to activation of reward systems in the human brain. The results of those studies may be relevant to pain management in humans, as basic animal research has shown that pharmacologic activation of reward systems can substantially reduce pain. Indeed, viewing pictures of a romantic partner was recently demonstrated to reduce experimental thermal pain. We hypothesized that pain relief evoked by viewing pictures of a romantic partner would be associated with neural activations in reward-processing centers. In this functional magnetic resonance imaging (fMRI) study, we examined fifteen individuals in the first nine months of a new, romantic relationship. Participants completed three tasks under periods of moderate and high thermal pain: 1) viewing pictures of their romantic partner, 2) viewing pictures of an equally attractive and familiar acquaintance, and 3) a word-association distraction task previously demonstrated to reduce pain. The partner and distraction tasks both significantly reduced self-reported pain, although only the partner task was associated with activation of reward systems. Greater analgesia while viewing pictures of a romantic partner was associated with increased activity in several reward-processing regions, including the caudate head, nucleus accumbens, lateral orbitofrontal cortex, amygdala, and dorsolateral prefrontal cortex – regions not associated with distraction-induced analgesia. The results suggest that the activation of neural reward systems via non-pharmacologic means can reduce the experience of pain.

## Introduction

The early stages of a new, romantic relationship can be a powerful and absorbing experience. Individuals in new romantic relationships report feeling euphoric and energetic. They also become emotionally dependent on, desire closeness with, and have highly focused attention on their partner [1]–[6]. Human neuroimaging studies have shown that feelings experienced during the early stages of a romantic relationship are associated with neural activations in several reward-system and affect-processing regions of the brain [1], [7], [8]. Those studies displayed pictures of participants' own romantic partners [9] to reliably evoke acute positive affect and self-reported feelings of love. In one such functional magnetic resonance imaging (fMRI) study, Aron and colleagues [1] instructed participants in new, romantic relationships to view pictures of their partner, and pictures of a familiar acquaintance who was the same age and sex as the participant's partner. Neural activations specific to viewing pictures of the romantic partner were observed in several reward-processing regions, such as the bilateral caudate nucleus and right ventral tegmental area. An earlier fMRI study using a similar protocol reported neural activations specific to the romantic partner pictures in reward regions such as the bilateral caudate nucleus and bilateral hippocampus [7]. The activation of reward structures caused by viewing pictures of a romantic partner has also been confirmed in a Chinese sample, suggesting the phenomenon may be culturally universal [8]. Collectively, these neuroimaging studies demonstrate that reward-system activation is a central component of self-reported feelings of love in new romantic relationships.

The engagement of reward systems by viewing pictures of a romantic partner is pertinent to the study of pain because several basic animal studies have shown reward-processing regions to be critically involved in analgesia [10]. For example, the nucleus accumbens and ventral tegmental area (two key reward processing structures) both play an important role in analgesic processes [11]–[14] – perhaps explaining why pleasurable and appetitive states such as sucrose consumption and anticipation of food reward reduce pain [15]. The results of those studies suggest that the activation of reward systems (perhaps even non-pharmacologically) could reduce pain in humans. Indeed, a recent behavioral study demonstrated that the presentation of romantic partner pictures is sufficient to reduce experimentally-induced pain [16]. The partner pictures reduced pain significantly more than when participants viewed pictures of a stranger or affect-neutral object, and the analgesic benefit was as strong as holding the partner's hand. The study was important in that it showed a mere representation of a romantic partner can reduce pain; however, the study was not designed to characterize the central mechanisms related to reward-induced analgesia.

Given recent behavioral results suggesting an analgesic benefit of rewarding experiences, and neuroimaging data showing those experiences to produce reward system activation, we hypothesized that analgesia during evoked feelings of love would be associated with reward system activation. Using fMRI of the human brain, we investigated the neurophysiologic substrates of analgesia produced by viewing pictures of a romantic partner.

## Materials and Methods

### Participants

Participants were 15 right-handed students (8 women and 7 men, age range 19–21 years, M = 20 years) in their first 9 months of a romantic relationship. All participants described themselves as intensely in love, and scored a minimum sum of 90 on the 9-point scale, 15-item short form of the Passionate Love Scale (PLS) [5].

### Experimental paradigm

All study procedures were approved by the Institutional Review Board at the Stanford University School of Medicine, and all participants provided written informed consent. Before arriving for the scan session, each participant provided three digital pictures of his or her romantic partner, and three pictures of an acquaintance of the same gender and attractiveness as the romantic partner. We sought to balance partner and acquaintance attractiveness because previous research has shown attractiveness to be associated with neural activations in reward areas [17]. Acquaintances were also individuals who the participants had known for approximately the same length of time as their partner and for whom the participant reported no romantic feelings. The attractiveness of both the romantic partners and acquaintances were rated independently (on a 0–10 numerical scale) by eight individuals who were blinded to the relationship type and who were not otherwise involved with the study. There was no difference in attractiveness of the acquaintances versus partners (*t*(28) = −0.35, *p* = 0.73). All pictures were cropped to display only the face.

At each participant's scan session, we first determined what temperature would produce moderate and high levels of pain. To determine temperatures used for moderate-pain and high-pain heat, participants were exposed to 15-second heat blocks, starting at 40 degrees Celsius (a non-painful temperature). Following each heat block, participants were asked to report the degree of pain on an 11-point visual scale (0 = no pain at all, 10 = worst pain imaginable). Each successive heat block was increased by 1 degree Celsius, until the participant reached their 10/10 (maximum) pain score. Then, the lower temperatures were retested to verify what temperatures elicited 4/10 (moderate) and 7/10 (high) pain. We presented thermal stimuli with a Medoc (Durham, NC) Advanced Thermal Stimulator 3×3 cm Peltier contact thermode. When in the scanner, the thermode was attached to the thenar eminence of the left hand, so that the right hand could be free for inputting pain ratings via a button box. The ramp rate for all trials (in and out of the scanner) was 10°C per second, requiring a maximum of 1.5 seconds to reach the target temperature.

While in the scanner, participants performed three distinct tasks: an acquaintance baseline condition, a romantic partner active condition, and a distraction control condition. In the *acquaintance* baseline condition, each participant was shown the pictures of his or her acquaintance via a projector and mirror display mounted on the head coil. Following the protocol of Aron and colleagues [1], participants were asked to focus on the picture and think about the displayed person. The use of the active baseline condition allowed us to separate neural activity specific to viewing pictures of a romantic partner from those of simply looking at an equally attractive and familiar face. In the *romantic partner* condition, participants viewed pictures of their partner, and were asked to focus on the picture and think about the person. Participants also underwent a *distraction* control condition. During the distraction trials, participants were asked to complete a word-association task that had been shown to effectively reduce pain in previous fMRI studies [18], [19]. The distraction control condition allowed us to determine whether or not the partner pictures were simply serving as a salient distractor from pain. In the distraction trials, a seed phrase was displayed (e.g., “Sports that do not use a ball”), and participants were instructed to silently think of as many responses as possible. The distraction task demands a high degree of attention, requires no movement, and is relatively free of an emotional component.

Each of the three conditions described above was performed under periods of no pain, moderate pain, and high pain. Participants were told that a range of temperatures would be presented, and were not told that only three discreet temperatures would be administered. All of the “no pain” presentations were given at a baseline temperature of 32 degrees Celsius. Moderate- and high-pain presentations used each individual's 4/10 and 7/10 temperature levels that were determined before the scan. Each condition (partner, acquaintance, and distraction) by pain (none, moderate, and high) combination was repeated 6 times, for a total of 54 randomly ordered trials. Following each trial, the participant rated his or her evoked pain, using the equipped button box and a projected visual analog scale. Pain ratings were collected immediately following (rather than during) the pain stimulus, so that task performance would be minimally affected by sensorimotor processing associated with rating pain on the response box.

Following the pain rating, participants completed a mental arithmetic count-back task for 13 seconds. This task (adapted from Aron and colleagues) [1] was designed to minimize emotional and sensory carryover between trials. In the task, participants were visually presented a 4-digit number, and were asked to count backwards by 7's as quickly and accurately as possible. The task was also part of the study manipulation, as participants were told their performance on the task was a central component of the experiment. The time course of each trial was as follows: trial ready cue (2 sec), acquaintance, partner, or distraction task with thermal stimulus (16 sec), pain rating (10 sec), and count-back (13 sec).

### Behavioral analysis and statistics

A single univariate ANOVA was performed to determine the effects of the partner and distraction tasks on self-reported pain. Two independent variables were entered into the model: heat pain level (no pain, moderate pain, and high pain), and condition (acquaintance, partner, and distraction). Pain ratings were entered as the dependent variable. Pain ratings were averaged over trial repetitions to yield a single pain score per subject, for each temperature by condition combination – with subject treated as a random effect.

After scanning was finished, participants completed a brief outtake form. To assess for possible demand characteristics, participants were asked, “What do you think was the purpose of this experiment?” Responses were evaluated to determine the number of participants who correctly determined the purpose of the study.

### fMRI data acquisition

We conducted scans at the Stanford University Lucas Center, using a 3T GE Signa system and 8-channel head coil. Functional blood oxygen level-dependent (BOLD) data were acquired using a T2*-sensitive spiral in/out pulse sequence [20]. Functional volumes consisted of 28 oblique (parallel to the AC-PC plane) slices covering the brain and brainstem (4 mm thickness, 0.5 mm gap, in-plane resolution 3.125×3.125 mm, repetition time = 2 sec, TE = 30 ms, flip angle = 90°, field of view = 20×20 cm). High-order shimming was performed before the functional scans [21]. A T1-weighted 3D-IR-FSPGR scan was acquired for anatomical reference (TE = 1.7ms, 124 slices, 1.2mm isotropic resolution).

### fMRI processing and analysis

Functional images were first corrected for cardiac and respiratory noise [22], and then realigned, resliced, and smoothed by 6mm, using SPM8 (Wellcome Department of Imaging Neuroscience, London). First-level statistics were performed on an individual level in native space. Condition-specific effects were estimated using a general linear model (GLM) approach. Conditions were described with a boxcar design and then convolved with the canonical hemodynamic response function. All phases of the scan protocol (cue, task, pain rating, and countback) were modeled, though only the task periods were used in contrasts. Statistical results maps for all planned contrasts were coregistered with the high-resolution structural images, normalized to MNI space, and resampled at a 1 mm isotropic voxel size using the DARTEL toolbox in SPM8 [23]. The spatially-normalized contrast maps were then used to conduct second-level (group) statistics with participant as a random effect. A grey matter voxel mask was applied to all second-level contrast maps.

Two major contrast analyses were performed. The first contrast identified neural activations and deactivations associated with viewing pictures of a romantic partner, while in pain, and controlling for both the effects of viewing pictures of an equally attractive acquaintance, and performing a distraction task. A conjunction analysis approach was used, which requires all identified voxels to demonstrate greater increase or decrease in activity compared to both the acquaintance and distraction tasks. The conservative approach (using the “conjunction” option in SPM8) ensured that all identified clusters were significantly different from both control conditions.

The second contrast identified neural regions associated with analgesia resulting from viewing pictures of a romantic partner, distinct from distraction analgesia. Analgesia (pain reduction) during the partner task was determined by subtracting pain ratings in the partner trials from pain ratings in the baseline acquaintance trials. Degree of analgesia was also calculated for the distraction task, by subtracting pain in distraction trials from pain in the acquaintance trials. To determine the neural responses specific to analgesia caused by viewing pictures of a romantic partner, analgesia was entered as a covariate in the second-level analysis, yielding a contrast map of all BOLD increases and decreases significantly associated with pain relief. The contrast map also masked out any significant BOLD responses associated with distraction analgesia, to identify only those analgesia responses specific to viewing pictures of a romantic partner. By reversing the distraction-analgesia mask, a separate map was also created to show analgesia-associated BOLD responses occurring in both the partner and distraction tasks.

All statistics were performed on the whole brain, with no region-of-interest or small-volume corrections. The group-level (mixed effects) contrast images were thresholded with a voxel-height significance threshold of *p*<.005 (uncorrected), requiring a *t*-value of 3.01. An FDR-corrected spatial extent threshold was not employed, because several reward and pain-modulatory nuclei have total structural volumes that are below FDR-corrected thresholds for the whole brain. For example, the ventral tegmental area has a volume below the FDR-corrected, *p*<.05 spatial threshold of approximately 271 mm^3^ [24]. Instead of an FDR correction, a spatial extent threshold of 64 contiguous voxels (a 64 mm^3^ region) was used to allow smaller regions to emerge in a whole-brain analysis. The use of combined height and spatial-extent thresholds in this manner has been demonstrated to provide a good balance between risk of Type I and Type II error [25]. However, even with the extent threshold we used, it is still possible that smaller reward structures (or specific regions of reward structures) would be too small to be identified.

## Results

### Behavioral

All participants scored highly on the self-reported scale of passionate love (mean = 109.8, SD = 11.2, range = 91.5–132.0), meeting the minimum required sum score of 90.

Table 1 presents pain ratings for the three tasks (acquaintance picture baseline, distraction control, and partner picture task), and three temperature levels (no pain, moderate-pain, and high-pain). The thresholding procedure was effective at determining temperatures to elicit 4/10 (moderate) and 7/10 (high) pain in the baseline condition. Averaging across all acquaintance baseline trials, pain during the moderate-intensity trials was rated at 3.7, and pain during the high-intensity trials was rated at 7.0.

**Table 1:** Pain ratings by temperature and task.

a)	None	Moderate	High
**Acquaintance**	0.1 (0.2)	3.7 (1.7)	7.2 (1.1)
**Distraction**	0.0 (0.0)	2.4 (1.5)	6.2 (1.6)
**Partner**	0.0 (0.0)	2.4 (1.8)	6.2 (1.7)

b)		Moderate	High
**Distraction**		1.3 (0.8) 36.0%	1.0 (1.1) 12.9%
**Partner**		1.4 (1.0) 44.7%	1.0 (1.4) 12.1%

**a)** Self-reported pain for none, moderate-intensity, and high-intensity heat (columns), and the three tasks: acquaintance baseline, distraction control, and partner active tasks (rows). Means are followed by standard deviations. **b)** Mean pain decrease during the distraction and romantic partner active tasks during moderate and high heat-intensity trials. Means and standard deviations (in parentheses) are provided, followed by average *percent* pain reduction.

A univariate ANOVA of pain ratings (with task and temperature as predictors) was performed. The two-predictor model strongly predicted pain ratings (adjusted R squared = 0.81). Temperature was a significant predictor of pain (*F*(2,14) = 276.95, *p*<0.0001). Bonferroni-adjusted post-hoc tests revealed that reported pain was significantly different between all levels of heat intensity (all pairwise contrast *p*'s<0.0001). Task was also a significant predictor of pain (*F*(2,14) = 4.72, *p* = 0.011). Bonferroni-adjusted post-hoc tests showed that pain was significantly reduced in both the distraction and partner conditions, contrasted with the acquaintance baseline condition (*p*'s = 0.026). There was no difference in pain between the distraction and partner conditions (*p* = 1.0). The “temperature by condition” interaction was not significant (*F*(4,14) = 1.07, *p* = 0.372). Because we were interested in neural mechanisms that generalize across pain levels, moderate- and high-intensity trials were aggregated for all neuroimaging analyses.

To determine the possible role of demand characteristics on self-reported pain, responses to the manipulation check were examined. A response was counted as a correct guess if the participant identified pain as the dependent variable (e.g., “to see how emotions affect pain”). Six out of the fifteen participants correctly guessed the purpose of the study.

### fMRI – BOLD responses during presentation of romantic partner pictures while in pain

The first group contrast identified the main effects of the partner task on neural responses during periods of moderate- and high-intensity pain. Identified clusters represent BOLD signal changes associated with the partner task that occurred over and above both the acquaintance and distraction tasks. The conjunction analysis (Table S1) revealed several regional areas of BOLD activity increase and decrease associated with viewing pictures of a romantic partner during pain.

**Figure 1:**
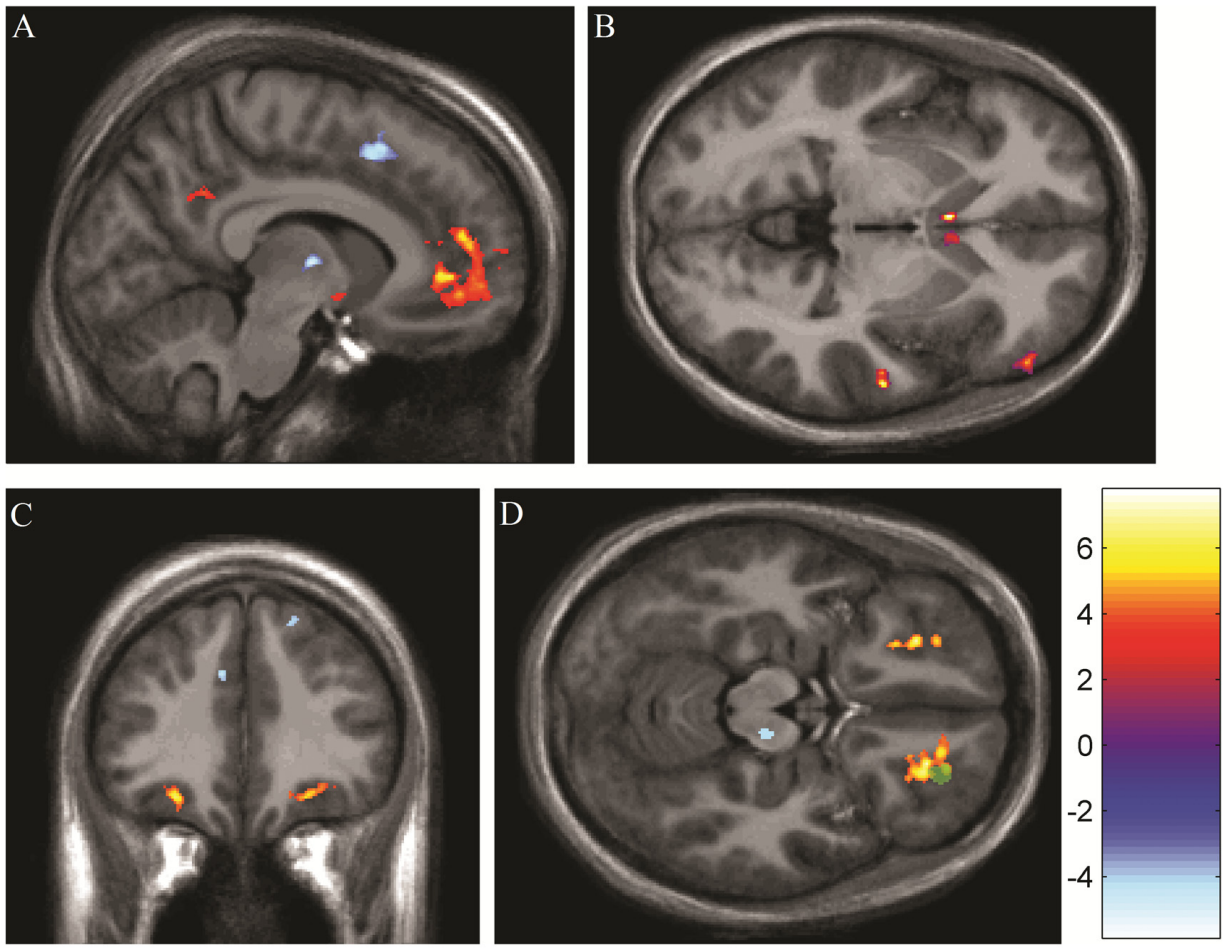
Neural responses associated with viewing pictures of a beloved during periods of acute experimental thermal pain. Significant clusters are shown on an MNI-normalized average of all participants' high-resolution structural scans. Neurological (right on right) convention is used. (a) Sagittal view (x = −8) of conjunction analyses showing areas of BOLD increase (yellow) and decrease (blue) associated with viewing pictures of a romantic partner, over and above both viewing pictures of an attractive and familiar acquaintance, and a word-association distraction task. Activations (anterior to posterior) include the medial orbitofrontal cortex, pregenual anterior cingulate cortex, amygdala, and precuneus. Deactivations are seen in the supplementary motor area and ventral lateral nucleus of the thalamus. (b) Axial view (z = −3) of neural activation associated with pain relief during viewing of romantic partner pictures. Greater pain relief was associated with greater activity in the right dorsolateral prefrontal cortex, bilateral caudate head, and right superior temporal gyrus. (c) Coronal view (y = 34) of neural activity increase (yellow) and decrease (blue) associated with pain relief during viewing the romantic partner pictures. Pain relief is associated with greater BOLD activity in the bilateral lateral orbitofrontal cortices, and decreased BOLD activity in the left dorsal anterior cingulate cortex and right supplementary motor area. (d) Axial view (z = −16) of neural activity increase (yellow) and decrease (blue) associated with pain relief during viewing the romantic partner pictures. Pain relief is associated with greater BOLD activity in the bilateral lateral orbitofrontal cortices, and decreased activity in the right brainstem, approximately in the location of the substantia nigra. Also shown (in green) is an overlapping area of the right lateral orbitofrontal cortex that was also positively associated with analgesia in the distraction condition.

The largest cluster of activation was found in the bilateral frontal cortex, projecting out from the pregenual anterior cingulate cortex, and extending into the medial orbitofrontal cortex (Figure 1a). Separate clusters of activation were observed in the subgenual anterior cingulate (BA 25) and mid-cingulate (BA 23) cortices, as well as the left precuneus (BA 31; Figure 1a), left amygdala (Figure 1a), and right hypothalamus.

Several BOLD activity decreases were also associated with viewing pictures of a romantic partner. Signal decreases were seen in the bilateral posterior insula, left thalamus (ventral lateral nucleus; Figure 1a), left inferior frontal cortex, right frontopolar area (BA 10), left supplementary motor area (Figure 1a), and right precentral gyrus (BA 6).

### BOLD responses associated with pain relief while viewing pictures of a romantic partner

The second group contrast assessed neural activity changes associated with analgesia produced by the romantic partner picture task. BOLD activity increases and decreases (Table S1) significantly correlated with pain relief during the partner trials were identified, masking out any regions also associated with pain relief in the distraction task.

BOLD activity in a number of regions was positively associated with pain relief in the partner task, including the bilateral caudate head (Figure 1b), bilateral nucleus accumbens, right dorsolateral prefrontal cortex (Figure 1b), right superior temporal gyrus (Figure 1b), bilateral lateral orbitofrontal cortex (Figures 1c and 1d), left amygdala, and right thalamus (ventral anterior nucleus).

Greater pain relief was associated with decreased BOLD activity in the right superior frontal gyrus (Figure 1c), left dorsal anterior cingulate cortex (Figure 1c), right brainstem (located in the substantia nigra and red nucleus region; Figure 1d), left anterior insula, right putamen, left supplementary motor area, and left parahippocampal area.

To determine if any BOLD responses were associated with analgesia in *both* the partner and distraction conditions, the distraction mask was reversed. Only one area of overlap was identified: the right lateral orbitofrontal cortex (green cluster in Figure 1d). Distraction analgesia (Table S2) was instead associated with activations in the left rostral anterior cingulate, left medial frontal gyrus, left middle frontal gyrus, bilateral putamen, left superior parietal cortex, right anterior cingulate cortex, left dorsolateral prefrontal cortex, left Broca's area, left orbitofrontal cortex (BA 47), and bilateral orbitofrontal cortex (BA 11).

## Discussion

In this study, we demonstrate that pain relief experienced while viewing pictures of a romantic partner is associated with reward system activation. We further show that the neural processes associated with reward-induced analgesia are distinct from those associated with distraction-induced analgesia.

Our first goal was to determine if viewing pictures of a romantic partner activates reward and limbic regions of the brain, even during periods of moderate- and high-intensity pain. We found a main effect for the romantic partner task on BOLD response in several reward and limbic regions. Viewing pictures of a romantic partner activated reward processing areas such as the amygdala (stimulus reward value learning) [26], hypothalamus (reward-stimulus associations) [27], pregenual anterior cingulate cortex (reward-related cognition) [28], and medial orbitofrontal cortex (hedonic experience processing) [29]. Several additional limbic regions, such as the precuneus, mid-cingulate, and subgenual anterior cingulate cortex were also associated with the viewing of romantic pictures. Because the contrast map controlled for both: 1) viewing pictures of an equally attractive and familiar acquaintance, and 2) distraction, the observed activations were likely specific to feelings evoked by the pictures of a romantic partner.

BOLD activity decreases were also observed with the romantic pictures task, and were localized mostly in pain-processing regions. Activity was suppressed in both the left and right posterior insula, areas responsible for sensory processing of pain [30]. BOLD activity was suppressed in the left ventral lateral nucleus of the thalamus, suggesting early-stage suppression of nociceptive signals [31]. Activity in the stimulus-contralateral premotor cortex was also suppressed, suggesting further reduction of pain processing [32]. Not all regions showing depressed BOLD activity were associated with classic pain-processing regions. Regions showing an activity decrease, but not typically connected to the pain experience, included the left inferior frontal cortex, and right frontopolar area.

As suggested by previous behavioral research, viewing pictures of a romantic partner effectively reduced self-reported pain [16]. We found that BOLD increases in several reward system regions were associated with greater pain relief during the partner task. Associations between pain relief and activations in the bilateral caudate head and nucleus accumbens were seen, identifying aspects of both the classic mesolimbic and nigrostriatal reward pathways. Furthermore, regions known to modulate reward processing, such as the amygdala, lateral orbitofrontal cortex, and dorsolateral cortex, were associated with pain relief. Many of those regions are commonly identified in reward-related neuroimaging tasks [33] and make up a corticostriatal network of reward processing [34], [35].

The corticostriatal reward network provides one route by which strongly valenced external cues may reduce the experience of pain. Cue-evoked reward prediction is known to involve orbitofrontal and dorsolateral prefrontal cortex activity in humans [36], [37], and both of those regions are strongly connected to reward-based evaluation and decision-making [38], [39]. In the case where pictures of a beloved may serve as a reward-related cue, activity in the orbitofrontal cortex or dorsolateral prefrontal cortex would modulate activity in mesolimbic and nigrostriatal reward systems via pronounced projections to the nucleus accumbens [39], [40] and caudate head [41]–[43]. Analgesia may then result from reward system projections to descending pain modulatory systems [44], which can inhibit ascending nociceptive messages at the spinal level [45]. The hypothesis that reward activation may suppress nociceptive processing at an early supraspinal or spinal stage is further supported by the wide range of pain processing regions that exhibited analgesia-correlated activity decrease. Pain relief produced by viewing pictures of a romantic partner was associated with suppressed activity in sensory (anterior insula and brainstem), affective (putamen, hippocampus, and anterior cingulate), and cognitive (supplementary motor area, superior frontal gyrus) aspects of the neural pain response network.

The reward-system activity we observed to be associated with pain relief during viewing of romantic partners was unlikely to be a general effect of analgesia, as an equal amount of pain relief evoked by a distraction task showed little engagement of reward systems. Distraction analgesia was associated with mainly cortical activations, and in many regions previously associated with the distraction task used [19]. The results suggest that there are multiple routes by which cognitive tasks can reduce pain, with emotion-based and distraction-based analgesia being two such possibilities. However, it is also true that the experience of pain relief itself can also serve as a rewarding experience. The nucleus accumbens [46] and amygdala [47] can be activated during various experiences of pain relief [48]. Placebo analgesia in particular has been associated with increased opioid transmission in a range of reward systems, including the orbitofrontal and dorsolateral prefrontal cortices, nucleus accumbens, and amygdala [49]. In the present study, pain relief resulting from both the partner pictures and distraction task activated an overlapping region of the right lateral orbitofrontal cortex. Therefore, the two types of analgesia were found to have largely separate, but somewhat overlapping, neural substrates.

Reward system activation may be one way in which analgesia systems are engaged, and neural overlap between the two systems is strong [50]. The relationship between reward processing and pain relief is supported by prior research showing analgesic benefits from pharmacologic manipulation of key reward systems [11], [13], [14]. It is possible that the analgesic benefit of a rewarding state confers a particular evolutionary advantage on organisms, including humans. The reduction of physical pain during the pursuit of a rewarding stimulus may allow individuals to pursue important goals even in the face of noxious and punishing stimuli, allowing an appetitive state to reduce the influence of aversive states on behaviors. Such inhibitory interactions between appetitive and aversive stimuli have long been reported in the psychological literature [51]. The engagement of reward systems provides one neurobiological route potentially underlying a number of recent findings, such as the presence or likeness of a partner reducing threat response [52] and pain [16].

While we have focused our discussion on reward systems, it is certainly true that the experience of viewing pictures of a beloved involves complex motivational, evaluative, memory, and other processes [2], [7], [53], [54]. Viewing pictures of a romantic partner is likely to be a more active process than the simple, passive experience of a rewarding state. While we attempted to control for active processes with the distraction task, it may not be possible to delineate and experimentally control all the aspects of the partner task. Several of the regions activated by the love task have been associated with other processes in fMRI tasks. The precuneus, for example, has been linked to episodic memory [55], perhaps indicating activation of memories linked to the picture. The region of the mid-cingulate we identified has been associated with visuospatial attention [56]. And, several of the regions identified (orbitofrontal cortex, hypothalamus, and amygdala) have also been observed during sexual arousal, especially in males [57].

Some methodological issues limit interpretation of the results. First, while participants retrospectively reported high attention to the tasks, there was no objective measure of task adherence or performance. Individuals paying close attention to the tasks would likely experience greater analgesia. It has been previously demonstrated that the suppression of neural pain processing activity is dependent on behaviorally-measured task performance [58]. Future studies may include a measure of task-attention (e.g., eye tracking). A second limitation is that the small sample size precluded the analysis of gender differences in the romantic partner analgesia effect. Third, demands characteristics could play a role in the observed analgesic responses. Six out of the fifteen participants correctly guessed the purpose of the experiment. If those individuals determined the purpose of the experiment early in the session, their self-reported pain may have been affected. Fourth, despite our attempts to control for attractiveness, it is likely that participants found their partner more attractive than their acquaintance. Some of the reward-processing regions we identified were also reported in a previous study examining BOLD response to attractive faces [17].

Surprisingly, we found no regions that showed *both* a main effect for the love task *and* a correlation with degree of analgesia. It is therefore not possible for us to determine what structure or system is critical for love-induced analgesia. The results also demonstrate that there is considerable individual variability in the analgesia experienced when looking at pictures of a beloved. The observed variability could be due to attention to the task, or could be a feature of the relationship (e.g., degree of obsession with the partner, or strength of the relationship).

Considerable advances in our understanding of pain and analgesia have been made in recent years, fueled to a great extent by emerging neuroimaging technologies. We show here that the activation of reward systems by viewing pictures of one's romantic partner is associated with reduced pain. A better understanding of these analgesic pathways may allow us to identify new targets and methods for producing effective pain relief.

## Supporting Information

Table S1 List of regions associated with viewing pictures of a romantic partner during pain. All significant clusters are seen in aggregated moderate- and high-pain trials. Parts (a) and (b) show main effects of the romantic partner task on regional BOLD increases and decreases, using conjunction analyses to control for both the acquaintance and distraction tasks. Parts (c) and (d) show BOLD changes that were significantly correlated with pain relief during the romantic partner task, controlling for distraction analgesia. Left is heat ipsilateral, right is heat contralateral. Reported clusters survived a voxel-level, uncorrected p<.005 (corresponding to a t-value of 3.01), and a cluster-level threshold of 64 contiguous voxels. The region name is listed, followed by coordinates (MNI), t-score at the peak voxel, p-value at the peak voxel, and cluster size. L = left, R = right, B = bilateral.
(0.05 MB DOC)

Table S2 List of regions associated with analgesia during a distraction task. All significant clusters are seen in aggregated moderate- and high-pain trials. Reported clusters survived a voxel-level, uncorrected p<.005 (corresponding to a t-value of 3.01), and a cluster-level threshold of 64 contiguous voxels. The region name is listed, followed by coordinates (MNI), t-score at the peak voxel, and cluster size. L = left, R = right, B = bilateral.
(0.04 MB DOC)
