# Supplementary material for: Viewing Pictures of a Romantic Partner Reduces Experimental Pain: Involvement of Neural Reward Systems
Source: PLoS One. 2010 Oct 13;5(10):e13309. doi: 10.1371/journal.pone.0013309 (PMC2954158; doi:10.1371/journal.pone.0013309)
Supplement: Table S1 — List of regions associated with viewing pictures of a romantic partner during pain. All significant clusters are seen in aggregated moderate- and high-pain trials. Parts (a) and (b) show main effects of the romantic partner task on regional BOLD increases and decreases, using conjunction analyses to control for both the acquaintance and distraction tasks. Parts (c) and (d) show BOLD changes that were significantly correlated with pain relief during the romantic partner task, controlling for distraction analgesia. Left is heat ipsilateral, right is heat contralateral. Reported clusters survived a voxel-level, uncorrected p<.005 (corresponding to a t-value of 3.01), and a cluster-level threshold of 64 contiguous voxels. The region name is listed, followed by coordinates (MNI), t-score at the peak voxel, p-value at the peak voxel, and cluster size. L = left, R = right, B = bilateral. (0.05 MB DOC) [file pone.0013309.s001.doc]

| Region | Coordinates (MNI) | *t* | *p* | Voxel count |
| --- | --- | --- | --- | --- |
| **a) BOLD activity increases:** |  |  |  |  |
| B orbitofrontal cortex | -3, +46, -2 | 7.72 | 0.000001 | 2539 |
| B pregenual cingulate cortex | -7, +42, -1 | 6.63 | 0.000006 | 1087 |
| L mid-cingulate (BA 23) | -3, -17, +31 | 4.53 | 0.000236 | 218 |
| R hypothalamus | +5, -3, -8 | 4.35 | 0.000333 | 84 |
| L precuneus (BA 31) | -6, -51, +31 | 3.90 | 0.008001 | 141 |
| L amygdala | -11, -2, -10 | 3.64 | 0.001339 | 89 |
| R subgenual anterior cingulate | +2, +20, -9 | 3.44 | 0.001992 | 220 |
| **b) BOLD activity decreases:** |  |  |  |  |
| R posterior insula | +45, -20, +16 | 7.24 | 0.000002 | 1182 |
| R frontopolar area (BA 10) | +31, +56, +26 | 5.64 | 0.000030 | 407 |
| L Supplementary motor area | -6, +17, +48 | 4.99 | 0.000099 | 665 |
| L posterior insula | -41, -20, +16 | 4.85 | 0.000129 | 1216 |
| R Precentral gyrus (BA 6) | +30, -6, +61 | 4.39 | 0.000308 | 266 |
| L inferior frontal cortex (triangular) | -39, +12, +21 | 4.20 | 0.000445 | 304 |
| L thalamus (ventral lateral) | -7, -12, +5 | 3.08 | 0.004075 | 132 |
| **c) increases associated with analgesia:** |  |  |  |  |
| L caudate head | -4, +11, -4 | 6.44 | 0.000008 | 85 |
| R superior temporal gyrus | +60, -14, -3 | 6.06 | 0.000015 | 126 |
| R dorsolateral prefrontal gyrus | +59, +30, +9 | 5.71 | 0.000027 | 289 |
| L lateral orbitofrontal gyrus | -26, +38, -16 | 5.56 | 0.000035 | 582 |
| R lateral orbitofrontal gyrus | +24, +38, -16 | 5.28 | 0.000058 | 404 |
| L amygdala | -19, -8, -11 | 4.66 | 0.000184 | 277 |
| R thalamus (ventral anterior) | +9, -6, +6 | 4.37 | 0.000320 | 96 |
| R caudate head | +5, +14, -4 | 4.19 | 0.000454 | 88 |
| L nucleus accumbens | -18, +14, -9 | 4.04 | 0.000608 | 124 |
| R nucleus accumbens | +14, +9, -6 | 4.03 | 0.000620 | 114 |
| **d) decreases associated with analgesia:** |  |  |  |  |
| L supplementary motor area | -6, +5, +71 | 5.45 | 0.000043 | 100 |
| R superior frontal gyrus | +25, +39, +52 | 4.61 | 0.000202 | 248 |
| R putamen | +33, +6, -6 | 4.31 | 0.000360 | 88 |
| L hippocampus | -28, -15, -24 | 4.16 | 0.000481 | 88 |
| L anterior insula | -28, +26, +10 | 3.78 | 0.001015 | 80 |
| L anterior cingulate | -8, +35, +32 | 3.85 | 0.000884 | 95 |
| R brainstem | +9, -22, -15 | 3.64 | 0.001339 | 125 |
