# Supplementary material for: Viewing Pictures of a Romantic Partner Reduces Experimental Pain: Involvement of Neural Reward Systems
Source: PLoS One. 2010 Oct 13;5(10):e13309. doi: 10.1371/journal.pone.0013309 (PMC2954158; doi:10.1371/journal.pone.0013309)
Supplement: Table S2 — List of regions associated with analgesia during a distraction task. All significant clusters are seen in aggregated moderate- and high-pain trials. Reported clusters survived a voxel-level, uncorrected p<.005 (corresponding to a t-value of 3.01), and a cluster-level threshold of 64 contiguous voxels. The region name is listed, followed by coordinates (MNI), t-score at the peak voxel, and cluster size. L = left, R = right, B = bilateral. (0.04 MB DOC) [file pone.0013309.s002.doc]

| Region | Coordinates (MNI) | *t* | Voxel count |
| --- | --- | --- | --- |
| L rostral anterior cingulate cortex | -8, +39, +12 | 4.28 | 394 |
| L medial frontal gyrus | -8, +29, +46 | 5.62 | 182 |
| L middle frontal gyrus | -36, +23, +42 | 4.27 | 883 |
| L putamen | -19, +4, +18 | 4.03 | 119 |
| R putamen | +21, +4, +13 | 3.58 | 120 |
| L superior parietal cortex (BA 7) | -32, -69, +43 | 4.17 | 776 |
| R anterior cingulate cortex (BA 32) | +12, +32, +14 | 3.48 | 132 |
| L dorsolateral prefrontal cortex (BA 10) | -26, +45, +16 | 3.47 | 70 |
| L Broca’s area (BA 45) | -60, +23, +3 | 4.21 | 149 |
| L orbitofrontal cortex (BA 47) | -40, +30, -18 | 4.56 | 115 |
| L orbitofrontal cortex (BA 11) | -32, +46, -17 | 4.69 | 73 |
| R orbitofrontal cortex (BA 11) | +24, +43, -16 | 3.01 | 141 |
|  |  |  |  |
